# Review: New feeds and new feeding systems in intensive and semi-intensive forage-fed ruminant livestock systems

**DOI:** 10.1016/j.animal.2021.100297

**Published:** 2021-12

**Authors:** J.M. Moorby, M.D. Fraser

**Affiliations:** a Institute of Biological, Environmental and Rural Sciences, Aberystwyth University, Gogerddan, Aberystwyth SY23 3EE, UK; b Pwllpeiran Upland Research Centre, Aberystwyth University, Cwmystwyth, Aberystwyth SY23 4AB, UK

**Keywords:** Animal production, Meat, Methane, Milk, Nutrient use efficiency

## Abstract

The contributions that ruminant livestock make to greenhouse gas and other pollutant emissions are well documented and of considerable policy and public concern. At the same time, livestock production continues to play an important role in providing nutrient-rich foodstuffs for many people, particularly in less developed countries. They also offer a means by which plants that cannot be digested by humans, e.g. grass, can be converted into human-edible protein. In this review, we consider opportunities to improve nutrient capture by ruminant livestock through new feeds and feeding systems concentrating on intensive and semi-intensive systems, which we define as those in which animals are given diets that are designed and managed to be used as efficiently as possible. We consider alternative metrics for quantifying efficiency, taking into account resource use at a range of scales. Mechanisms for improving the performance and efficiencies of both individual animals and production systems are highlighted. We then go on to map these to potential changes in feeds and feeding systems. Particular attention is given to improving nitrogen use efficiency and reducing enteric methane production. There is significant potential for the use of home-grown crops or novel feedstuffs such as insects and macroalgae to act as alternative sources of key amino acids and reduce reliance on unsustainably grown soybeans. We conclude by highlighting the extent to which climate change could impact forage-based livestock production and the need to begin work on developing appropriate adaptation strategies.

## Implications

Livestock will continue to play an important role in future food security strategies for much of the global population. At the same time, climate change is a significant threat to the human population, and livestock agriculture has been a prominent source of greenhouse gases. New feeds and feeding systems offer potential for ruminant production to improve nutrient use efficiency at both individual animal and system levels, thereby reducing associated greenhouse gas emissions. Ruminant agriculture must also start to adapt to rising global temperatures, and the continued development of new feeds and forages will play a key part in this.

## Introduction

A growing global human population, an increasing global demand for meat and milk as societies become more affluent, and the contributions, directly and indirectly, that livestock production make to greenhouse gas (**GHG**) and other pollutant emissions are all well documented (Steinfeld et al., 2006, Godfray et al., 2010) and of considerable policy, media and public concern. Despite these concerns, ruminant livestock and their products will continue to play a significant role in providing nutrient-rich food for many people, particularly in less developed countries. They also offer a means by which plants which cannot be digested by humans, e.g. grass, can be converted into human-edible protein, and thus provide a means for producing food in areas unsuitable for cropping due to poor soil or climatic conditions. The pressing challenge is therefore to identify opportunities to maximise the efficiency of use of ruminant feed and minimise related environmental footprints.

The development of new feeds and new livestock feeding systems has been an ongoing process for centuries. Standard systems for determining basic livestock feed values (proximate analyses) were first developed in the mid-1800s (at the German Weende Experiment Station) followed by the publication of livestock feeding standards in the late 19th Century and early- to mid-20th Century (see Coffey et al., 2016). The Welsh Plant Breeding Station (originally part of the University College of Wales, Aberystwyth) was founded in 1919 with the remit of improving livestock agriculture in Britain, and related outputs transformed grasslands and grassland science internationally (Moore-Colyer, 1999); this development work continues today in Aberystwyth. While many of the societal challenges faced in 1919 continue today (food security, rural depopulation, poor financial sustainability of, e.g. upland farming), plant breeding in general remains tasked with addressing global environmental crises, including climate change and loss of biodiversity. This article will focus on potential next steps for forage-fed ruminant livestock systems. Although there is an argument for reducing the global numbers of ruminants (e.g. Ripple et al., 2014), with the use of appropriate technological innovations, strategies, and resources, ruminant livestock could continue to play a significant role in providing food within environmentally sustainable agricultural systems (Tedeschi et al., 2015).

**Fig. 1:**
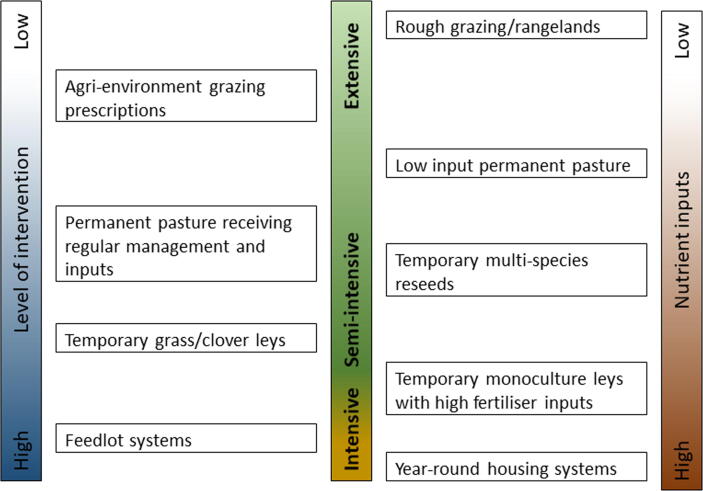
Mapping of the central components of alternative ruminant livestock systems to definitions of ‘intensive’, ‘semi-intensive’ and ‘extensive’ production; where temporary leys are <5 years old, and permanent pasture are >5 years old.

Given that we are considering ruminant production systems, for this article, we have assumed that forage, likely grass-based, will form the main dietary component. We recognise that there is a continuum of production types (Fig. 1), and define intensive livestock systems as those in which animals are given a diet that is designed to be used as efficiently as possible. A complete diet is one that is produced and presented to the animals in a way that minimises their ability to select out individual components, and can be formulated to maximise the partitioning of feed nutrients into productive outputs and minimise potentially polluting emissions. Such diets would typically be those offered to housed animals, and may include total mixed rations and compound concentrate feeds that include a variety of ingredients. Semi-intensive systems include those in which diet choices are less limited, but are still designed to support efficient production. These include grazing leys sown with specific forage species and cultivars that are managed to optimise the efficiency of use of feed resources, or areas of longer-term pastures that are managed to improve their nutritional characteristics using applications of fertiliser and cutting regimes (Fig. 1). This review will consider recent advances in feeds and feeding systems for intensive and semi-intensive grassland-based systems, i.e. systems with the greatest nutrient demands and levels of intervention, highlighting priority areas for future research.

## Measuring animal performance and associated opportunities for improvement

One of the first considerations when appraising livestock systems is the choice of metric to be used to gauge improvements in performance (Table 1). The simplest definitions of livestock production efficiency, such as feed conversion efficiency (e.g. kg feed in/kg product out) or individual animal efficiencies of use of energy (e.g. MJ product energy produced/MJ feed energy consumed) or nitrogen (**N**), show that ruminant livestock (beef and dairy cattle, sheep and goats) are relatively inefficient compared to monogastrics (pigs and poultry) (Herrero et al., 2013). However, to judge ruminant livestock only by these metrics ignores the abilities of these animals to use human-inedible feeds to produce highly nutritious food for people. The digestive systems of pigs and poultry mean that they typically need to be fed diets containing a higher proportion of feeds that could be consumed by humans than ruminant livestock (Wilkinson, 2011), and thus the feed conversion efficiency of production of human-edible foods can be similar, or even better for ruminants than monogastrics. When only minimal amounts of human-edible protein are included in animal feeds, ruminant livestock can be highly efficient at converting human-inedible feed to human-edible meat and milk, particularly when offered forage-based diets (Wilkinson, 2011, Wilkinson and Lee, 2018).

**Table 1:** Definitions of efficiency of food production from livestock (of any species), as determined by units of comparison and the scale at which comparisons are made.

Efficiency term	Typical metric used	Applicable scales
Feed conversion efficiency	kg feed DM intake/kg product	Individual animal
Feed nitrogen (N) use efficiency	kg N in product/kg feed N intake	Individual animal
Efficiency of human-edible protein production	kg human-edible protein/kg feed protein intake	Individual animal
Efficiency of human-edible food production	kg human-edible product/kg human-edible food	Individual animal, farm
Feed energy use efficiency	MJ product energy/MJ feed energy intake	Individual animal
Land use efficiency	kg product/ha agricultural land kg product/ha land that could be used for human food production	Farm, region, country, global
Farm energy use efficiency	kg product/MJ whole farm energy input	Farm
Emission intensity	g pollutant output/kg product	Individual, farm, region, country, global
Life cycle assessment	kg product/unit of burden	Farm, region, country, global

More recently, new metrics of efficiency, based on losses from the system rather than production gains, have become commonly used. Livestock are responsible for approximately 14.5% (in CO_2_-equivalents) of global anthropogenic GHG emissions, of which about 44% is methane (Gerber et al., 2013). Ruminant livestock produce significant amounts of methane as a by-product of microbial fermentation, and measures of GHG emission intensities (kg CO_2_-equivalent/kg product output) show these are also consistently higher for ruminants than monogastrics (Ripple et al., 2014). Livestock production also contributes directly and indirectly to nitrous oxide emissions from manure and slurry management (Brown et al., 2020) and via the use of N fertilisers and the deposition of N in urine patches (Chadwick et al., 2018). Nitrogen excreted from livestock also contributes significantly to undesirable nitrate leaching to groundwater and atmospheric ammonia emissions (Webb et al., 2005, Backes et al., 2016), and the latter, with other oxides of N, contribute to poor air quality through the formation of secondary particulate matter (Leip et al., 2015). Despite being relatively efficient at extracting energy from sources that monogastric animals cannot use effectively (i.e. plant fibres), ruminant livestock are relatively inefficient at using dietary protein for productive purposes (g N in product/g N intake) (Calsamiglia et al., 2010). Dairy cows are generally intensively managed, yet approximately three quarters of the N they consume are excreted rather than being converted into milk protein (and growth in younger lactating animals) – the mean efficiency of feed N use for milk protein production by individual animals was found to be about 25% in North American cows and about 28% in north European cows (Huhtanen and Hristov, 2009), with these values decreasing as feed protein intake increases above animal requirements. However, a wide range of individual animal efficiencies (between approximately 14% and 45%) highlights the potential to improve the capture of feed protein into milk with appropriate and accurate balancing of the animals’ feed. Use of technologies such as mid-infrared spectroscopy, already incorporated into some milking machines, could be used to monitor individual animal production efficiency and health by measuring milk composition (Gengler et al., 2016) and dynamically altering nutrient intakes through the provision of different feeds to tailor, for example, the protein and energy supplies to match the requirements of that animal.

In an extensive review of potential animal management mitigation options to reduce GHG emission intensities from livestock operations, Hristov et al. (2013) concluded that the most effective method is an increase in individual animal productivity, allowing a reduction in animal numbers while maintaining edible product output. It has long been known that ruminant livestock emit methane as a by-product of the fermentation process, and efforts to reduce methane production began decades ago, albeit for somewhat different reasons to those which prevail today. Until the late 1970s, methane excretion from ruminant animals was largely considered a waste of potentially useful energy that could otherwise be used for productive purposes. Blaxter and Clapperton (1965) demonstrated the positive relationship between feed energy digestibility and methane output in cattle and sheep fed to maintenance and just above, but also highlighted the negative relationship between the two parameters when animals were fed at three times maintenance. These findings suggest that daily methane emissions will be greater from individual high-producing ruminant animals fed higher quality (i.e. more digestible) diets. However, those animals fed to have feed intakes at multiples of maintenance energy requirements will be more efficient and excrete less methane per unit of energy intake, leading to lower emission intensities (i.e. lower yields of methane per unit of productivity) (e.g. Gerber et al., 2011). Thus, intensifying to maintain system yields from fewer animal would lead to overall lower pollutant outputs.

The ability to ferment fibrous materials to extract energy-yielding nutrients (in particular volatile fatty acids) is a key benefit of ruminant livestock compared to monogastrics. Much of the protein that ruminants digest in the small intestine is microbial protein formed in the rumen, the production of which depends on the efficiency of fermentation (Stern and Hoover, 1979, Hoover and Stokes, 1991, Bach et al., 2005). This complicates the precision with which ruminant livestock can be fed an appropriate balance of amino acids compared to pigs and poultry, because in the latter, protein digestion mostly occurs in the digestive tract before any fermentation occurs. The supply and balance of amino acids that are absorbed from the gut and made available to productive tissues (i.e. muscles and mammary glands) are what ultimately determines the efficiency of growth (Whittemore et al., 2001, Siegert and Rodehutscord, 2019) and milk production (Schingoethe, 1996, Lapierre et al., 2006). Thus, Dijkstra et al. (2013) suggested that the greatest scope for improving the efficiency of use of dietary protein in cattle is via ensuring optimum supplies of rumen degradable N plus optimising the efficiency of N use for the synthesis of protein.

Improvements in milk production by high-yielding dairy cows can be achieved by supplying protein sources that are not completely degraded in the rumen, thus by-passing the processes of feed N being incorporated into microbial protein (e.g. Santos et al., 1998). Many feeds are by-products of human food (and fuel) production, such as dried distillers’ grains and rapeseed meal, while others, including soybeans, may be grown specifically for livestock feed. Obtaining accurate values for the qualities of soybeans and their by-products is difficult because of the complexities of supply chains. However, approximately 673 thousand tonnes of whole soybeans and 2.23 million tonnes of soybean meal and cake were imported into the UK in year July 2019 to June 2020 (Agriculture and Horticulture Development Board, 2020b). Over the same period, approximately 1.16 million tonnes of soybean meal and cake were used for animal feeds in Great Britain (Agriculture and Horticulture Development Board, 2020a) and Northern Ireland (Department of Agriculture, Environment and Rural Affairs, 2020), suggesting that about 42% of possible supplies of soybean meal and cake was used for other purposes, such as for human food and other products (including biodiesel from soya oil). The amino acid balance of soya protein makes it an important feed (and human food) ingredient, although land use change driven by its production in South America is well documented (Carvalho et al., 2019). Reducing the use of soya grown and used as an animal feed is a key objective to increase the sustainability of livestock production, and this will require both the development of replacement protein sources and reductions in the amount of protein fed to livestock.

## Measuring system performance and associated opportunities for improvement

Part of the lowering of emission intensity of ruminant products with higher quality diets can be attributed to changes in rumen fermentation if higher diet quality is achieved through the provision of starchy feeds (cereal grains) as a replacement for high-fibre feeds. Fermentation of starches tends to result in the promotion of biochemical pathways within the rumen that lead to greater production of propionic acid, and a lower production of hydrogen, which would be scavenged by methanogens within the rumen microbial population to produce methane. However, the ability to use high-fibre feeds, many of which are produced as a result of the production of human food or which can be grown in places that human foods do not grow well (e.g. grass v cereals), has always made ruminant livestock a valuable component of agriculture. Mottet et al. (2017) recently calculated that 86% of livestock feeds used globally (for both monogastric and ruminant animals) are not human edible, typically comprising grassland and by-products of human food production. In addition, a significant proportion of land used globally for grassland production, about 57%, cannot be converted to crop production for growing human food (Mottet et al., 2017). Although reducing cattle numbers to decrease their use of human-edible foods has been calculated as having the potential to significantly reduce agricultural GHG emissions, particularly methane emissions (Jayet et al., 2020), this analysis did not include permanent pastures and appeared to treat all concentrate feeds as equally negative in terms of food energy use by livestock. While it is true that some land currently used for livestock production could be used for human food production (setting aside the financial and societal costs of doing so), a significant proportion of agricultural land in parts of the United Kingdom and other European countries has been classified as less favoured due to constraints relating to abiotic factors such as soil type, topology and climate. Schader et al. (2015) showed that forage-fed ruminants could help maintain food security at the same time as reducing the environmental impact of livestock production by utilizing resources that could not be used for human food production.

Differences in the way livestock are fed between, e.g. the United States, in which grazed grass is used primarily for beef production, with relatively little used for other livestock types (Peters et al., 2014), and, e.g. north-west Europe, where grazed grass is used more widely for dairy cows (although this is in decline as more animals are kept permanently housed; Schils et al., 2019, van den Pol-van Dasselaar et al., 2020), mean that measures of system efficiency can also be very different depending on the metrics used to judge it. Life cycle assessment (LCA) is an approach that enables more holistic and objective comparisons between different systems, allowing investigation of trade-offs and downstream effects and impacts. Styles et al. (2018) calculated that intensification of dairy production in the UK could increase the carbon footprint of milk production if beef production from the dairy herd decreases, as might happen if sexed semen is used to only breed replacement heifers, indicating that the wider implications and trade-offs of changing production systems need to be considered. Similarly, an initial LCA of milk production in Costa Rica by Mazzetto et al. (2020) calculated that specialist dairy farms, compared to dual-purpose farms producing both milk and beef, produce milk with the lowest environmental footprint when considering the production system from birth to farm-gate. However, expanding the system boundary showed that dual-purpose dairy-beef farms had the lowest emission of GHG to produce 1 kg of milk and 100 g beef.

Another reason the carbon footprint of UK milk production may increase is through a move to annual crops such as maize silage (Styles et al., 2018). Perennial crops, such as permanent pasture, have lower cultivation requirements following establishment, and reduced land tillage that minimises soil carbon losses. Soteriades et al. (2018) used LCA methods to show that choice of grazing system – using genetically improved forage grasses – coupled with efficient manure management could reduce the environmental burden of milk production in the UK significantly, achieving up to 22% and 40% reductions in eutrophication and acidification potentials, respectively. For housed animals, several manure management options exist to mitigate the emissions of pollutants (e.g. Petersen et al., 2013), but these can be expensive to implement and are not as useful for grazing livestock. In comparison, careful choice of grazing materials offers a relatively quick and cheap way of improving the efficiency of livestock production. However, a lack of robust data, especially for calculating carbon footprints of agricultural products, hampers the ability to identify the best pollution mitigation options, which in turn will impede the UK’s ambition to be net zero by 2050. Despite recent research efforts to support improved resolution of UK enteric methane emissions reporting to the International Panel on Climate Change (Ricci et al., 2013, Moorby et al., 2015, Ferris et al., 2017), the volume of emission data for ruminants at pasture is only a fraction of that collected under controlled conditions. Likewise, much more research is required to characterise temporal changes in soil organic carbon under different forage crops and grassland management systems.

## Opportunities to improve forage-based system nutrient use efficiencies

Reducing the use of unsustainably grown feed commodities such as soya and palm oil is a key objective for improving the sustainability of livestock production. However, for this to be achieved, alternative feeds need to be found to partially or wholly replace these valuable dietary ingredients to prevent land use change that can occur to grow them, as global livestock production continues to increase. Kingston-Smith et al. (2013) highlighted the role of forage plant breeding to produce more home-grown feeds, particularly for ruminant livestock. While key plant breeding targets have focussed on parameters such as biomass yield, disease resistance and persistency, the only nutritional trait of forages to be included in current UK national list trials is D-value (digestibility of the organic matter expressed as a proportion of the DM; see Finch et al., 2014). Although D-value is an important characteristic, other nutritional characteristics are equally or even more important.

**Fig. 2:**
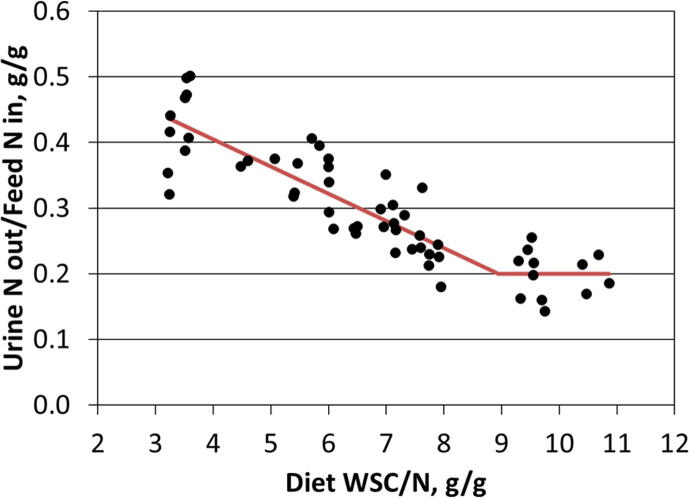
Relationship between diet ratio of water-soluble carbohydrate (WSC) and nitrogen (N) concentrations and the proportion of feed N intake excreted in urine in dairy cows fed fresh ryegrass-based diets. Dots represent the mean values for individual animals measured and the solid line represents a split line regression (R^2^ = 0.77) with the breakpoint of 8.94 g WSC/g N in the complete diet (Moorby, 2014).

A key component of fresh grasses is the water-soluble carbohydrate (WSC) fraction. Although increasing the dietary concentration of WSC by the use of certain perennial ryegrass (*Lolium perenne*) varieties has led to improvements in milk yield from dairy cows (Miller et al., 2001), an arguably more important finding is the reduction in apparent excretion of N in urine from cows when they are fed high-WSC ryegrass-based diets (Miller et al., 2001, Moorby et al., 2006), likely as a result of increasing the efficiency of rumen N use in both fresh (Lee et al., 2002) and ensiled (Merry et al., 2006) forages. Simple changes in management practices, such as the allocation of fresh strips of grass pastures in the afternoon, rather than in the morning, capitalise on allowing plants to photosynthesise during the day, resulting in higher concentrations of WSC. Such changes have been shown to increase milk yields from dairy cows (Orr et al., 2001) and improve weight gain in growing cattle (Gregorini et al., 2006) and can influence the concentration of urinary N (Vibart et al., 2017, Beltran et al., 2019), which in turn could lower the daily emissions of ammonia and nitrous oxide from grazed pastures. Analysis of data collected from whole-body N partitioning experiments with dairy cows fed fresh grass-based diets has shown that excretion of N in urine, expressed as a proportion of feed N intake, is minimised when the whole-diet ratio of WSC to N is greater than approximately 9 g/g (Moorby, 2014) (Fig. 2). Below this value, there is a negative relationship between the dietary WSC to N ratio and apparent excretion of feed N in urine. Increasing feed WSC above this value results in no further reduction in urine N excretion, possibly because the absorption of ammonia-N from the rumen is minimised along with its subsequent excretion in urine.

Another way of reducing N pollution is to reduce N inputs, although this must be done in ways that allow productivity to be maintained. In grazing systems, N inputs are often crucial to maintaining pasture productivity (although the value of clovers and other legumes in fixing atmospheric N is increasingly recognised – see below). However, Peyraud and Astigarraga (1998) highlighted the negative relationship between grass WSC and CP concentrations – as more N fertiliser is applied, grass CP concentrations increase and WSC concentrations decrease. Thus, although there is a strong correlation between N fertiliser application and grass N concentration, there is relatively little effect of grass N fertiliser applications on the eventual supply of usable protein to the small intestine (Peyraud and Astigarraga, 1998). This is likely through a change in the efficiency of use of feed N in the rumen for microbial growth, with higher concentrations of grass N being captured less efficiently with lower concentrations of WSC.

Leguminous forages offer significant sustainability benefits for forage-based livestock systems, reducing the requirements of synthetic fertiliser inputs and improving ruminant livestock productivity (Lüscher et al., 2014). However, their high N, but low WSC, concentrations typically lead to relatively low rates of N use efficiency unless combined with feed ingredients that help improve N use efficiency, such as grasses bred for elevated concentrations of WSC (Merry et al., 2006, Moorby et al., 2009). Some legumes contain secondary compounds that improve N use efficiency by slowing the rate of proteolysis. Red clover (*Trifolium pratense*) contains the enzyme complex polyphenol oxidase, which has been shown to reduce protein degradation within the silo as well as the rumen (Lee, 2014). Polyphenol oxidase catalyses the formation of quinones in fresh material, which bind to proteins, thereby reducing protease and lipase activities. Reducing or slowing the rate of proteolysis of forage proteins (Hart et al., 2016) can increase the efficiency of use of feed proteins for productive purposes (Marita et al., 2012). Condensed tannins are present in some agronomically important forages such as bird’s-foot trefoil (*Lotus corniculatus*) and sainfoin (*Onobrychis viciifolia*), and these bind to proteins in the rumen to slow the rate of proteolysis and reduce the excretion of urine in N when compared to forages such as ryegrass or lucerne (*Medicago sativa*) that do not contain tannins (Brinkhaus et al., 2016, Huyen et al., 2016, Rufino-Moya et al., 2019). Consumption of forages containing condensed tannins has also been linked lowering of methane yields (Min et al., 2020) although results can be variable over time (Duval et al., 2016). There is a risk that at high concentrations, tannins become an antinutritive factor, leading to poor utilisation of dietary protein (Fraser et al., 2000). Other plant secondary compounds that have proven to improve feed use efficiency include phytoestrogens in red clover. Although these can negatively impact fertility in breeding ewes (Kelly and Shackell, 1982), the growth rate of lambs finished on swards of red clover with higher concentrations of formononetin tends to be higher, with heavier carcasses produced, compared to lambs finished on pastures of low formononetin red clover or ryegrass (Moorby et al., 2004).

## Alternative feeds and feeding systems

Highly productive ruminant livestock on forage-based diets require additional supplementation to perform to their genetic potential. A large proportion of concentrate ingredients may comprise by-products of human food production, such as oil seed meals, straws, and spent brewers’ and distillers’ grains, the use of which as valuable livestock feeds (Halmemies-Beauchet-Filleau et al., 2018) avoids alternative methods of disposal such as landfill or composting. However, cereals and other ingredients such as soybeans are frequently used to improve dietary energy supply and protein or amino acid balance. To reduce the livestock industry’s reliance on the use of feeds that are grown specially for feeding to livestock, alternative feeds need to be identified and developed.

The fibre content of diets, and specifically the NDF and lignin concentrations, has long been used in ration formulation as a predictor of feed intake and fibre degradability in ruminants, particularly dairy cows (Mertens, 2010). However, relatively low correlations between cereal grain yield and the ability to ferment the straws to release sugars (Garrido et al., 2018) suggest there is potential to simultaneously select for both in future breeding programmes. Likewise, the variability of lignin in particular cereal components, e.g. oat husks, has long been known (Welch et al., 1983). This has been targeted by recent breeding activities to reduce this and thereby improve the feed value of oat husks for ruminant livestock (A.A. Cowan, pers comm). Husks constitute about a quarter of the oat grain, so improving the rumen fibre digestibility of the husk by reducing its lignin concentration would allow the use of a higher proportion of the oat grain to be used for productive purposes by livestock (Winfield et al., 2007).

The search for more efficient livestock systems has also led to interest in less conventional feedstuffs. Macroalgae (seaweeds) have been used to feed livestock for many centuries, either by direct grazing in coastal locations or by collection and drying for later feeding (Makkar et al., 2016). The nutritional value for ruminant livestock varies considerably depending on the type (red, brown, green) and species of seaweed and seasonal changes. In particular, the mineral content of various species makes them a useful supplement, but this can also limit the amounts that can be fed. Similarly, the cell wall and storage carbohydrates of some seaweeds include cellulose and starch, but others are rich in other polysaccharides such as xylans, carrageenans, fucoidans and laminarin (Makkar et al., 2016), which require adaptation of the rumen microbial population to be degraded effectively. Microalgae of various types and species also offer potential use as livestock feed supplements, being valuable sources of protein, fatty acids, and minerals. Their composition can vary depending on cultivation conditions, but as yet production methods and costs currently limit their widespread use in livestock diets (Madeira et al., 2017). However, there is growing interest in the use of some seaweeds to reduce the emissions of methane from livestock when fed them (McCauley et al., 2020). This is the latest in a long line of supplements and feed additives that have been evaluated as potential means of reducing GHG emissions from ruminants (Cottle et al., 2011, Cobellis et al., 2016). Bromoform, which is a key bioactive compound in some seaweeds that can reduce the enteric production of methane in ruminants (Abbott et al., 2020), is toxic, which may limit their use in milk production if significant quantities of bromoform are secreted in milk. However, there is no evidence that concentrations of bromoform in milk from dairy cows fed *Asparagopsis armata* would be unsafe for human consumption even at a ‘high’ rate of diet inclusion of 1% of dietary organic matter (Roque et al., 2019). At 0.5% and 1% rates of dietary inclusion methane emissions (g/d) were significantly reduced from the cows that were fed the seaweed, compared to control animals, part of which was explained by substantial reductions in feed intake, although methane yield (g methane/kg feed DM intake) was also reduced. There is more work to be done to provide evidence that reductions observed in laboratory-based trials and in limited numbers of animal experiments are translated into practical and consistent effects *in vivo* (Jayanegara et al., 2012).

Another novel feedstuff currently receiving research and mainstream media attention is insects. Despite the high nutritional value of many insect types, cultural acceptance of eating them directly by humans has a number of barriers to overcome in many countries (Rumpold and Schlüter, 2013b, Kim et al., 2019), but using insects as a protein source for livestock (Sanchez-Muros et al., 2014) is likely to be more favourably received by consumers. Life cycle assessment of production of insect protein as a replacement for human food or livestock feed suggests that more work is needed to fully understand the systems and improve the potential environmental sustainability of them, particularly in terms of energy use (Smetana et al., 2016). Current European regulation generally prohibits the use of substrates of animal origin such as manure and waste food for growing insects, as it does for traditional livestock production (European Food Standards Agency Scientific Committee, 2015) and to achieve good yields of insect biomass for direct human consumption, high quality feedstocks would need to be used (Smetana et al., 2016). Good insect biomass yields could be obtained from substrates such as dried distillers’ grains with solubles or beet pulp, although both of these are valuable and already widely used ruminant livestock feeds, so their alternative use would generate competition. Nevertheless, there is potential to use poorer grade substrates, such as cattle manure (Hussein et al., 2017) to grow insects and generate useful feed ingredients for livestock to consume and thus produce more traditional and, to many consumers, more acceptable products (meats and milk) should regulations on origins of substrates for their growth change.

**Fig. 3:**
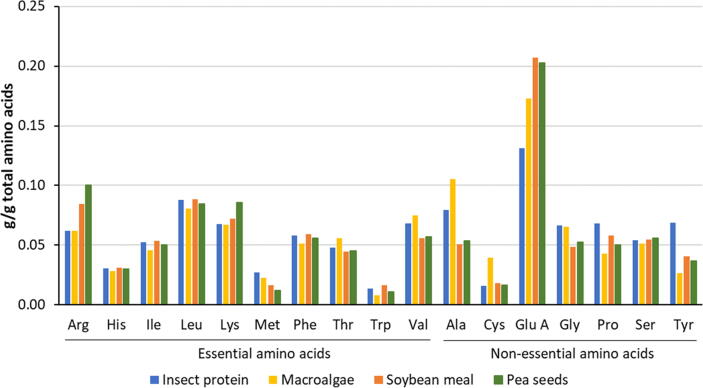
Mean amino acid profiles of CPs (g/g total amino acids) from insects (Rumpold and Schlüter, 2013a), macroalgae (seaweeds) (Makkar et al., 2016), soybean meal (Feedipedia, 2020b) and pea seeds (Feedipedia, 2020a). The mean sum of amino acids in CPs was 78.4, 75.0, 86.1 and 83.9 g/100 g for insects, macroalgae, soybean meal and pea seeds respectively.

A particular area where alternative feeds could play an important future role is in the supply of amino acids. One reason for the relatively high efficiency of use of dietary proteins by pigs and poultry compared to ruminant livestock is the ability to formulate and supply diets with high amino acid scores. In ruminant animals, degradation and utilisation of dietary proteins by the rumen microbial population complicate precise formulation, although many recent models and ration formulation systems for dairy cows incorporate predictions of the supply and requirements of amino acids for production (Schwab and Broderick, 2017). Soybean and soybean meal are used extensively in livestock diets because of their high protein concentration, useful amino acid profile, high digestibility and low fibre concentration. Alternatives such as peas, which can be grown in places that soya cannot (such as the UK) have a similar amino acid profile to soya (Fig. 3), and offer a more sustainable home-grown protein source. Insect proteins may offer a similar source of amino acids for livestock (Fig. 3) allowing systems to be developed that do not compete for land and other resources used directly for human food production. Similarly, pressure on good quality land used to produce feed for livestock could be eased by the use of other resources, such as coastal waters and other aquatic areas. Despite relatively high concentrations of CP in some seaweeds, the amino acid profile of most species is regarded as being deficient for most ruminant livestock, with the exception of sulphur-containing amino acids (methionine and cysteine) (Makkar et al., 2016), although there can be significant seasonal variability in total amino acid concentrations (Gaillard et al., 2018). Some microalgae, such as the cyanobacteria *Arthrospira sp.,* have a high protein concentration and a good essential amino acid profile, comparable to that of soya (Madeira et al., 2017), although the nutritional composition heavily depends on cultivation conditions. As such, assuming efficient methods can be developed to produce it, microalgae may offer an excellent source of protein that could help increase the efficiency of livestock production.

## The future of livestock systems

Looking to the not-too-distant future, climate change will inevitably lead to a step change in ruminant livestock production systems which must be prepared for now. Although rising temperatures have direct effects on the animals (Fodor et al., 2018), a potentially more serious consequence will be the effect on forage production. Thus, alterations in water availability and temperature increases may require a significant change in which forage species are grown. In temperate regions of the world, cool season grasses dominate pasture-based agriculture. These are typically C3 plants, such as ryegrasses (*Lolium* spp.), fescues (*Festuca* spp), timothy (*Phleum pratense*) and cocksfoot/orchard grass (*Dactylus glomerata*), and recent UK summers have demonstrated the vulnerability of these to dry conditions (Arshad and Fraser, 2020). In warmer environments, warm season and tropical grasses (typically C4 plants) such as signalgrasses (*Urochloa* spp*.* and hybrids), Rhodes grass (*Chloris gayana*) and Napier grass (*Cenchrus purpureus*) are commonly used. Forage maize (*Zea mays*) is a C4 plant of tropical origin that has been successfully bred to enable its cultivation at increasingly northerly latitudes, and while maize silage is a useful feed for ruminant production due to its high concentration of starch, it is an annual crop that is typically harvested in late autumn leaving bare soils prone to erosion. *Miscanthus* spp*.* is another C4 plant that has been more recently successfully introduced to Europe as a perennial biomass crop. Although its nutritional value is greatly inferior to Napier grass, its morphology and propagation are similar, and so its introduction to the UK and northern European countries has proved the principle that Napier grass or other tropical forage plants could be exploited as part of adaptation to climate change in these regions. Given that it generally takes forage breeding programmes around 15 years to produce a new, performance-tested variety for inclusion on recommended lists, research initiatives in this area are required urgently. Without viable, predictable, low-cost forages, grassland-based livestock production will become untenable for many, and given the growing need to avoid conflicts with land use for human food or bioenergy production, switching to more concentrate-based diets is likely to be unacceptable to policymakers or consumers.

## Conclusions

Livestock will continue to play an important role in food security for much of the global population. Climate change is a significant challenge to the global human population, and while livestock production continues to contribute to GHG emissions, more efficient livestock production feeds and feeding systems can offer ways to minimise these. At the same time, forage-based livestock systems will have to adapt to a changing climate and the impacts that this will have on their primary feedstuffs. This will likely require the adoption of new feeds and forages, which should now be the focus for research.

## Ethics approval

Not relevant.

## Data and model availability statement

Not relevant.

## Author ORCIDs

**JMM:** https://orcid.org/0000-0002-4449-8432

**MDF:** https://orcid.org/0000-0003-3999-1270

## Author contributions

**JMM**: Conceptualization, writing – original draft preparation.

**MDF**: Writing – reviewing and editing.

## Declaration of interest

None.
